# A Systematic Review on Renal and Bladder Dysfunction after Endoscopic Treatment of Infravesical Obstruction in Boys

**DOI:** 10.1371/journal.pone.0044663

**Published:** 2012-09-13

**Authors:** Pauline M. L. Hennus, Geert J. M. G. van der Heijden, J. L. H. Ruud Bosch, Tom P. V. M. de Jong, Laetitia M. O. de Kort

**Affiliations:** 1 Department of Urology, University Medical Center Utrecht, Utrecht, The Netherlands; 2 Department of Epidemiology, Division Julius Center for Health Sciences and Primary Care, and Department Otorhinolaryngology, Division Surgical Specialties, University Medical Center Utrecht, Utrecht, The Netherlands; 3 Pediatric Renal Center Wilhelmina Children's Hospital and University Medical Center Utrecht and Elisabeth Children's Hospital and Academic Medical Center Amsterdam, Amsterdam, The Netherlands; University of Sao Paulo Medical School, Brazil

## Abstract

**Background:**

Posterior urethral valves (PUV) may cause subtle to severe obstruction of the urethra, resulting in a broad clinical spectrum. PUV are the most common cause of chronic renal disease in boys. Our purpose was to report the incidences of kidney and bladder dysfunction in boys treated with endoscopic valve resection for PUV.

**Methodology:**

We searched MEDLINE and EMBASE databases until 1st of July 2011, to identify original papers that described outcome of endoscopic valve resection (EVR) in boys. We extracted information on (1) patient characteristics and clinical presentation of PUV related to outcomes and (2) the post-treatment absolute risks for kidney and bladder dysfunction.

**Principal findings:**

Thirty-four studies describing renal function, vesicoureteral reflux (VUR), incontinence, and urodynamic bladder function after EVR in 1474 patients were retrieved. Patients treated for PUV show high percentages of chronic kidney disease (CKD) or end stage renal disease (ESRD), 22% (0–32%) and 11% (0–20%), respectively. Elevated nadir serum creatinine was the only independent factor associated with renal failure. Before treatment, VUR was present in 43% of boys and after EVR, VUR was present in 22%. Post treatment, 19% (0–70%) was reported to suffer from urinary incontinence. Urodynamic bladder dysfunction was seen in many patients (55%, 0–72%) after treatment of PUV.

**Conclusions:**

The reported cumulative incidence of renal and bladder dysfunction in patients with PUV after endoscopic PUV treatment varies widely. This may reflect a broad clinical spectrum, which relates to the lack of a standardised quantification of obstruction and its severity. Moreover, the risk of bias is rather high, and therefore we put little confidence in the reported estimates of effect. We found elevated nadir serum creatinine as a predictor for renal dysfunction. In order to be able to predict outcomes for patients with PUV, an objective classification of severity of obstruction is mandatory.

## Introduction

Posterior urethral valves (PUV) are obstructing membranous folds within the lumen of the posterior urethra, forming the most common cause of congenital urethral obstruction in male children [1], [2]. PUV may cause subtle to severe obstruction of the urethra, resulting in a broad clinical spectrum, with variable dysfunction of the urinary tract. PUV are the most common cause of chronic renal disease in boys [1], [2]. In the developed world, an increasing number of PUV cases are identified by prenatal ultrasonography. Primary valve ablation is considered to be the treatment of choice for PUV [3], [4]. We found many case series on the outcomes of boys after endoscopic valve resection over the past years. Due to the broad clinical spectrum of PUV, the outcome of endoscopic valve resection (EVR) may vary widely. There are no previous systematic reports on long-term outcomes of lower and upper urinary tract function after primary valve ablation. Our purpose was to report the kidney-, bladder dysfunction, complications and additional surgery for post-treatment follow-up in boys with EVR for PUV and to study the relation of these outcomes with patient characteristics and clinical presentation of PUV.

## Materials and Methods

### Search Process

This study was conducted using the PRISMA (Preferred reporting items for systematic reviews and meta-analyses) guidelines (table S1 – PRISMA 2009 checklist) [5]. A literature search of PubMed and EMBASE was performed on July 1st 2011. Various synonyms were used for infravesical obstruction and endoscopic treatment. We combined our topical search strategy with synonyms for children to exclude irrelevant studies (table S2 – Search strategy).

**Figure 1:**
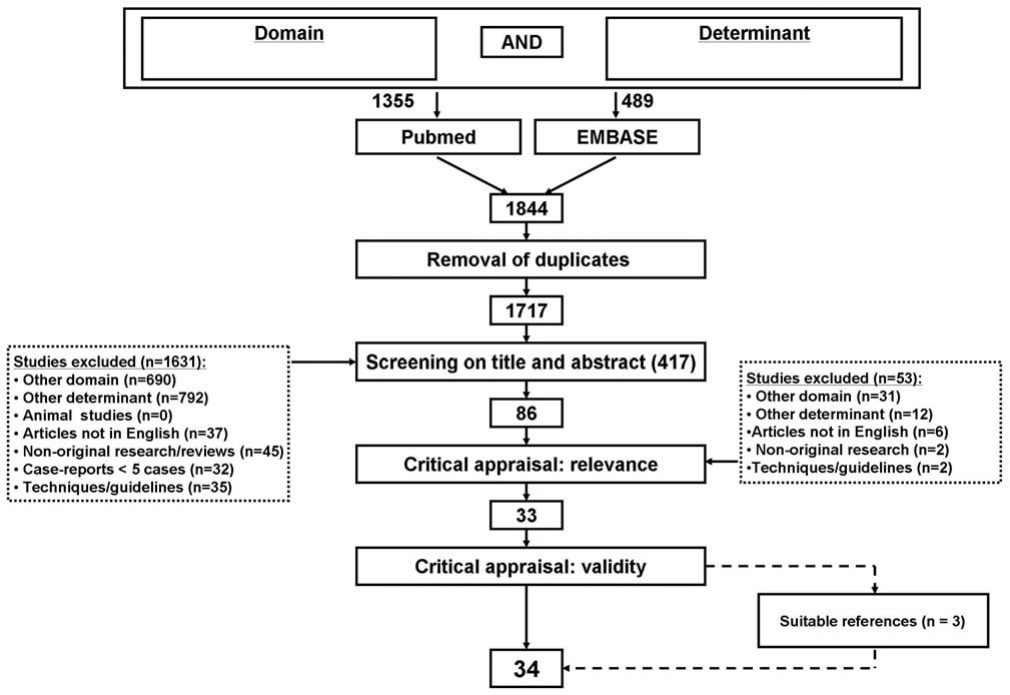
Flow-chart.

Two investigators (P.H. and L.d.K) independently screened the title and abstracts of all the retrieved articles using predefined selection criteria. They selected studies that included boys with EVR for infravesical obstruction, for which original follow-up data for at least one conventional clinical outcome was reported. Only studies reporting outcome of primary EVR without other surgical interventions: e.g. vesicostomy or ureterocutaneostomy, were included. The complete flowchart is presented in figure 1. All discrepancies were resolved by discussion. Hence, results are based on full consensus. We excluded non-English articles, studies with five or less children, articles describing point of techniques, review articles and animal studies.

Thereafter we assessed the risk of bias, notably due to lack of randomized and concealed allocation, similarity of treatment, blinding and completeness of data of the selected studies for each of the clinical outcomes. We extracted the outcome data for renal and bladder dysfunction. Articles that did not meet the selection criteria, or had more than 20% missing data were excluded. To retrieve possibly omitted studies we checked references of included and related articles. We extracted information on patient characteristics and clinical presentation of PUV considered related with outcomes and the post-treatment absolute risks for kidney and bladder dysfunction. We tabulated these data to look for patterns to identify specific subgroups according to clinical presentation with different results for selected outcomes.

### Data Extraction and Principal Endpoints

Data were extracted by two independent investigators (P.H. and L.d.K) from the full-text article of each included study. All discrepancies were resolved by discussion. Hence, results are based on full consensus. The principal outcomes were renal function, vesicoureteral reflux (VUR), incontinence, urodynamic findings,complications and additional treatments. These outcomes were chosen because of their clinical importance and frequency of reporting. When not given we (re-)calculated means and percentages from the reported data. For some outcome data we summed results over studies.

### Outcomes

The events of interest were renal function, VUR, urodynamic findings, incontinence and postoperative complications and additional procedures. The criteria for outcomes of renal function are chronic kidney disease (CKD), and end stage renal disease (ESRD). *CKD* was defined as a glomerular filtration rate (GFR) <60 mL/min per 1.73 m^2^ body surface area or creatinine values more than two times expected for age. *ESRD*, also known as CKD stage 5, was defined as GFR<15 mL/min per 1.73 m^2^ body surface area or the need for renal replacement therapy. If no definition could be retrieved from the text, that outcome was not included. GFR was estimated according to the method of Schwarz et al. [6] Serum nadir creatinine was defined as lowest measured creatinine value. Elevated creatinine is defined as more than twice the normal value for their respective age. If other definitions were used, that definition is defined in table 1. The criterion for *VUR*, and *VUR resolution* had to be based on findings on voiding cystourethrogram (VCUG) or direct isotope cystogram preferably according to the system proposed by the International Reflux Study Committee [7].

**Table 1:** Renal function.

*Title*	*Pts*	*Mean Age*	*FU months*	*Elevated nadir creatinine*	*Postoperative renal function*		
	*(N)*	*months (range)*	*Mean (range)*		CKD	ESRD	Total CKD & ESRD
Deshpande 2011 [30]	14		3 days	114.7 µmol/L^a^	N/A	N/A	N/A
			12 months	56.1 µmol/L			
Sarhan 2011 [26]	120	24 (0–180)	43^b^ (24–192)	43/120 (36%)^c^	26/120 (22%)	18/120 (15%)	44/120 (37%)
Ansari 2010 [31]	227	30 (0–192)	86 (6–192)	↑ 39/227^d^ (17%)	69/227 (30%)	27/227 (12%)	96/227 (42%)
Kibar 2010 [32]	13	45 (12–108)	N/A	↑ 4/13^e^ (31%)	N/A	0/13 (0%)	N/A
Sarhan 2010 [9]	120	24 (1–180)	53 (24–144)	1.0 mg/dL a	26/120 (22%)^e^	18/120 (15%)	44/120 (37)
Uthup 2010 [15]	30	3 (0–72)	93 (60–192)	↑ 20/30^f^ (63%)	4/30 (13%)	6/30 (20%)	10/30 (33%)
Sarhan 2008 [33]	65	0	82^b^ (12–172)	N/A	N/A	6/65 (9%)^c^	N/A
Godbole 2007 [34]	31	21	60 (3–120)	N/A	N/A	5/31 (16%)	N/A
Kajbafzadeh 2007 [16]	38	22 (0–66)	54 (24–84)	N/A	2/38 (5%)^c^	1/38 (3%)	3/38 (8%)
Androulakakis 2005 [17]	18	<3	112 (72–204)	10/18 (56%)^g^	4/18 (22%)^g^	2/18 (11%)	6/18 (33%)
Schober 2004 [12]	70	90 (24–168)	25 (1–78)	0/70 (0%)	0/70 (0%)	0/70 (0%)	0/70 (0%)
Lopez Pereira 2003 [35]	16	<3	144 (64–290)	↑ 9/16 (56%)	6/16	N/A	N/A
Podesta 2002 [10]	8	11 (1–35 )	139	1.6 mg/dL^a^	2/8^d^ (25%)	1/8 (13%)	3/8 (38%)
Puri 2002 [19]	38	37 (0–126)	61 (2–150)	↑ 12/38^d^ (32%)	12/38^d^ (32%)	N/A	12/38^d^ (32%)
De Gennaro 2001 [20]	11	<3	66	N/A	N/A	1/11 (9%)	N/A
Minimberg 1989 [36]	32	48	N/A	N/A	N/A	3/32 (9%)	N/A
**Mean**					**151/685 (22%)**	**88/783 (11%)**	**218/669 (33%)**

FU = follow up, N/A = not available.

CKD = chronic kidney disease: glomerular filtration rate (GFR)<60 mL/min per 1.73 m^2^ body surface area or creatinine values more than two times expected for age.

ESRD = end stage renal kidney disease: GFR<15 mL/min per 1.73 m^2^ body surface area or the need for renal replacement.

a Mean.

b Median.

c Definition CKD creatinine >2 mg/dL for more than 1 month.

d Definition elevated serum creatinine: >1 mg/dL.; Definition CKD: creatinine >1.6 mg/dL.

e Definition elevated serum creatinine >0.56 ml/dL.

f Elevated creatinine was not defined.

g Definition elevated serum creatinine and CKD: creatinine >88 µmol/L.

We used terminology defined by the International Continence Society (ICS) as a standard for outcomes of bladder dysfunction [8]. When other terminology was used in an included article, we appointed these to the recent designation if the same requirements were met. *Dryness* was defined as completely dry both day and night with no need to wear pads. Criterion for estimated bladder capacity (BC) for age was preferably calculated by the formula: (age×25)+25 mL or (age×30)+30 mL. A BC of more than 20% difference from expected BC for age was defined as an *increased* respectively *decreased BC*.

*Low bladder compliance* was defined as detrusor pressure higher than 10 cm H_2_O at expected bladder capacity for age. *Bladder hypocontractility* was defined as maximal voiding detrusor pressure (Pdetmax) lower than 20 cm H_2_O. *Post void residual* (PVR) was defined as a residual of more than 20% of expected BC. *Detrusor overactivity* had to be clearly defined, according to the International Continence Society preferably with pressures above 15 cm H_2_O [8]. We report c*omplications* and *additional surgery* the way they were defined in the articles.

## Results

### General

Results of our search strategy are shown in figure 1. We have identified 1844 titles, of which 1803 studies did not meet the selection criteria or were duplicate publications retrieved from the different databases. After the review of 41 full-text studies, 31 publications remained that met all inclusion criteria, and 3 articles were retrieved by reference checking.

In total, 34 studies were included with 1474 patients eligible for analysis.

Time of surgery ranged from 0 to 180 months. The follow-up ranged from 1 to 290 months.

A formal risk of bias assessment, notably for information and selection bias, was hampered due to limitations in the design and reporting of the included studies. Studies provide estimates of effects (cumulative incidences). The risk of bias is rather high, for most studies standardization and blinding of outcome measurements were absent, while the risk of selective reporting of the more severe patients was high. Moreover, for many the completeness of outcome data was uncertain. Because of these potential limitations it may be assumed that there is significant distortion of the reported results, while the variability in design and reporting limited the comparison of studies.

### Renal function

(See table 1.) Renal function is the most reported outcome in all included studies. The number of patients with an elevated creatinine before treatment is given in six articles and ranges from 17 to 57%. Two studies reported a mean creatinine on admission of 1.0 mg/dL and 1.6 mg/dL [9], [10]. Not all articles reported both, some only reported a total number of patients with renal function loss. Five articles report an association ofof elevated nadir serum creatinine with the future development of CKD or ESRD. Mean follow-up time ranged from 12 to 144 months. The mean percentage of patients with CKD at the end of follow-up was 22% (5–32%). Mean percentage of ESRD was 11% (0–20%) Data of the total number of boys with CKD or ESRD could be extracted from nine articles, mean 33% (8–42%).

### Vesicoureteral reflux

(See table 2.) In most patients, VUR tended to disappear after EVR. Before treatment with EVR, VUR was present in 43% (22–67%) of boys and 33% (18–65%) of ureteral units (UU). After EVR, VUR was present in 22% (10–38%) of boys, respectively 12% (6–26%) of UU. Severity of VUR preoperatively was reported in six out of eight studies. Most studies (4 out of 6) more often reported higher VUR grades than lower ones. Three studies only reported high grade VUR.

**Table 2:** Vesico-ureteral reflux.

*Title*	*Pts*	*Mean age*	*Mean follow-up*	*VUR preoperatively*		*VUR preoperatively*	*VUR post-operatively*	
	*(N)*	months (range)	months (range)	*Cases*	*UU*	*Low grade/high grade (N)*	*Cases*	*UU*
Kajbafzadeh 2007^a^ [14]	8	65 (24–84)	54 (24–84)	3/8 (38% )	3/16 (19% )	3/0	1/8 (13% )	1/16 (6%)
Kajbafzadeh 2007 [16]	50	22 (0–66)	54 (24–84)	16/50 (26%)	18/100 (18%)	0/16	5/50 (10%)	6/100 (6%)
Androulakakis 2005 [17]	18	<3	112 (72–204)	7/18 (39%)	9/36 (25%)	N/A	N/A	3/36 (8%)
Priti 2005 [37]	20	15^b^	6	12/20 (60%)	19/40 (48%)	6 UU/17 UU	N/A	4/40 (10%)
Kim 1996 [38]	20	30 (0–180)	106	11/20 (55%)	26/40 (65%)	7/4	6/18 (33%)	N/A
Nonomura 1999 [13]	74	60(3–192)	N/A	39/74 (53%)	64/148 (43%)	28/11	28/74 (38%)	N/A
Kurth 1981 [39]	124	<1: 13	N/A	31/124 (25%)	47/248 (19%)	35 UU/12 UU	20/123 (16%)	20/246 (8%)
		1–3 yr: 24			Grade I-IIa: 25			
		4–9 yr: 69			Grades IIb-IV: 22			
		>10 yr: 18						
Johnston 1979 [40]	66	N/A	(12–240)	44/66 (67%)	65/132 (49%)	N/A	N/A	34/132 (26%)
**Mean**				**163/381 (43%)**	**251/762 (33%)**		**60/277 (22%)**	**68/574 (12%)**

N/A = not available.

VUR = Vesicoureteral reflux; UU = ureteral units.

Low grade VUR: grade I–III; High grade VUR: grade IV–V.

a Only boys with anterior urethral valves included.

b Median.

### Incontinence

(See table 3.) Eleven studies investigated dryness after valve ablation. Four out of 11 studies reported preoperative incontinence in 48% of boys (12–100%). One study [11] only included boys presenting with incontinence and PUV, 4 studies [1], [12]–[14] included patients irrespective of incontinence prior to surgery. After EVR, the incidence of incontinence was reduced to 28% (0–70%) during a follow up of 6 months to 4.5 years. Postoperative daytime incontinence was found in 13% (7–35%) of boys and nighttime incontinence in 25% (5–70%); 22% (range 0–70%) of boys had nighttime and/or daytime incontinence.

**Table 3:** Incontinence.

*Title*	*Pts*	*Mean Age*	*Pre-operative incontinence*	*Follow-up*	*Post-operative incontinence*		
	*(N)*	*months (range)*	*N(%)*	*mean months (range)*	*Total DT/NT N(%)*	*DT N(%)*	*NT N(%)*
Nakamura 2010 [11]	20	141 (84–156)	20/20 (100%)	6	14/20 (70%)	7/20 (35%)	14/20 (70%)
Kibar 2010 [32]	13	45 (12–108)	N/A	86.4	2/13 (15%)	N/A	N/A
Uthup 2010 [15]	30	3 (0–72)	N/A	60	N/A	N/A	14/30 (47%)
Sarhan 2008 [33]	55	0	N/A	82^a^	12/55 (22%)	N/A	3/55 (5%)
Godbole 2007 [34]	14	21	N/A	12^a^ (36–90)	5/14 (36%)	4/14 (29%)	4/14 (29%)
Kajbafzadeh 2007 [16]	50	22	6/50 (12%)	54 (24–84)	0/50 (0%)	N/A	N/A
Schober 2004 [12]	70	90 (24–168)	47/70 (67%) NT	25 (1–78)	28/70 (40%)	3/70 (4%)	25/70 (36%)
			33/27 (47%) DT				
			31/70 (44%) NT+DT				
Podesta 2002 [10]	8	11	N/A	128 (61–205)	6/8 (75%) age 5 yr	N/A	N/A
					1/8 (12.5%) age 11.6		
Nonomura 1999 [13]	74	60	26/74 (35%)(incl. 15, 20% NT)	N/A	20/74 (27%)	9/74 (12%)	11/74 (15%)
Pieretti 1993 [1]	36	(0–108)	22/36 (61%)	24	7/36 (20%)	N/A	5/36 (14%)
Nijman 1991 [41]	85	N/A	N/A	>60^b^	5/85 (6%)	N/A	N/A
**Mean**			**121/250 (48%)**		**94/435 (22%)**	**23/178 (13%)**	**76/309 (25%)**

N/A = not available.

NT = night time incontinence.

DT = day time incontinence.

a median.

b Age at last follow-up.

### Urodynamic findings

(See table 4. )Seventeen papers reported on urodynamic studies after valve ablation. Urodynamic study was done one month to 20 years after EVR. Urodynamic abnormalities were found in 55% (0–72%). In seven studies, a decreased bladder capacity (BC) was found in 42%, (14–60%). A mean of 29% (0–50%) boys had poor bladder compliance and 31% (0–64%) had detrusor overactivity. Hypocontractile bladder was seen in 35% (0–73%), reported in eight studies. PVR was found in 31% (0–56%) [10], [12], [14]–[22]. Four studies reported on two or more urodynamic studies during follow-up. Taskinen et al. reported a small BC for age of 23 mL one month after EVR and a large BC for age, 112 mL, one year after treatment. One study reported that detrusor overactivity was found in 46% of patients 6 months after valve ablation, decreasing to 25% 4.5 years after valve ablation [16]. A decrease in detrusor overactivity was also found in two studies, showing 57% at 5 years of age decreasing to 20% at 9 years of age after EVR [23] and 73% in children younger than 12 decreasing to 0% in children older than 12 year old [20]. In these three studies bladder hypocontractility increased during follow-up ranging from 0–27% to 21–71%. The percentage of patients with PVR increased from 4 to 29% during 4.5 years follow-up and 0% to 43% 12.5 years after EVR.

**Table 4:** Urodynamics.

*Title*	*Pts (N)*	*Mean age months ( range)*	*UDS postop (months)*	*Poor bladder compliance*	*Detrusor overactivity*	*Hypocontr. bladder*	*Decreased BC*	*Increased BC*	*% PVR*
Ansari 2011 [42]	25	30 (24–60)	3	13/25 (52%)	5/25 (20%)	3/25 (12%)	15/25 (60%)	N/A	N/A
Sarhan 2011 [26]	48	24 (0–180)	43^a^ (24–192)	17/48 (46%)	21/48 (60%)	N/A	13/48 (36%)	9/48 (24%)	N/A
Uthup 2010 [15]	30	3^a^	No UDO	N/A	N/A	N/A	N/A	N/A	9/30 (30%)^b^
Taskinen 2009 [43]	25	0.5^a^ (0–10)	1	N/A	N/A	N/A	Mean BC 23 ml	-	N/A
			12	N/A	N/A	N/A	-	Mean BC 112 ml	N/A
Godbole 2007 [34]	7	21	N/A	4/7 (57%)	N/A	N/A	1/7 (14%)	N/A	N/A
Kajbafzadeh 2007^c^ [44]	8	65 (24–84)	6	0/8 (0%)	0/8 (0%)	0/8 (0%)	N/A	N/A	0/8 (0%)
Kajbafzadeh 2007 [14]	24	22 (0–66)	Pre-treatment	N/A	11/24 (46%)	0/24 (0%)	N/A	N/A	1/24 (4%)
			6	N/A	11/24 (46%)	0/24 (0%)	N/A	N/A	1/24 (4%)
			54	N/A	6/24(25%)	5/24 (21%)	N/A	N/A	7/24 (29%)
Androulakakis 2005 [17]	18	<3	149	N/A	N/A	10/18 (56%)	5/18 (28%)	N/A	10/18 (56%)
Schober 2004 [12]	70	90 (24–168)	25 (1–78)	N/A	N/A	N/A	N/A	N/A	18/70 (26%)
Emir 2002 [18]	26	54 (2–156)	54 (2–156)	13/26 (50%)	10/26 (38%)	N/A	15/26 (58%)^d^	N/A	8/26 (31%)
Podesta 2002 [10]	8	11 (1–35)	139	1/8 (12.5%)^e^	1/8 (12.5%)	N/A	N/A	N/A	1/8 (12.5%)^b^
Puri 2002 [19]	38	37 (0–126)	NA	11/38 (29%)^a^	N/A	N/A	11/38 (29%)	N/A	14/38 (37%)
De Gennaro 2001 [20]	11	<3	≤12 yr age	N/A	8/11 (73%)	3/11(27%)	1/11 (9%)	5/11(45%)	0/11 (0%)
			>12 yr age		0/7 (0%)	5/7 (71%)	1/7 (14%)	2/7 (29%)	3/7 (43%)
De Gennaro 2000 [23]	30^f^	6	62 (first)	2/30 (7%)	17/30 (57%)	0/30 (0%)	N/A	N/A	N/A
			112 (last)	1/30 (3%)	6/30 (20%)	15/30 (50%)	N/A	N/A	N/A
De Gennaro 1998 [21]	11	N/A (most <6)	118 (96–156)	2/11 (18%)	2/11 (18%)	3/11(27%)	N/A	N/A	1/6 (17%)
Krishna 1998 [22]	6	72–144)	6	3/6 (50%)	N/A	N/A	3/6 (50%)	N/A	3/6 (50%)
De Gennaro 1996 [45]	65		74	15/65 (23%)	25/65 (39%)	24/65 (37%)	N/A	N/A	N/A
Kim 1996 [38]	20	7^a^ (0–180)	48^a^ (1–240)	7/20 (35%)	8/20 (40%)	N/A	N/A	N/A	N/A
**Mean**				**87/292 (30%)**	**91/276 (33%)**	**68/192 (35%)**	**63/168 (38%)**	**16/66 (24%)**	**75/245 (31%)**

UDS = urodynamic study.

N/A = not available.

Poor bladder compliance = detrusor pressure >10 cm H_2_O at expected bladder capacity (BC) for age.

Bladder hypocontractility = maximal detrusor pressure (Pdetmax)<20 cmH_2_O.

Decreased BC = BC>20% smaller than expected BC for age.

Increased BC = BC>20% larger than expected BC for age.

PVR = post void residual: >20% of expected BC for age.

a Median.

b Definition PVR: >10% of expected BC for age.

c 8 boys with anterior urethral valves.

d <90% of calculated capacity.

e poor compliance defined as <15 mL/cm H_2_O.

f Undergoing 86 UDS (mean 2.8 each) with an interval of at least 3 years between first and last examination.

### Complications and additional surgery after primary treatment

(See table 5.) Strictures after endoscopic valve resection are reported in five of the included articles, ranging from 0 to 3.6% with a follow-up ranging from 3 months to 21 years. Lal et al [24] was the only study reporting the incidence and predisposing factors for postfulguration urethral structures as main research objective, they found that a urethral stricture developed in 3.6%.

**Table 5:** Complications & Additional Surgery.

*Title*	*Pts (N)*	*Mean Age (range, months)*	*FU (months)*	*Strictures (%)*	*Additional surgery (%)*	*Additional treatment*
Sarhan 2011^a^ [26]	120	24 (0–180)	43^b^ (24–192)	N/A	18/120 (15%)	Repeat valve ablation
Kajbafzadeh 2007 [16]	50	22 (0–66)	54 (24–84)	0/50 (0%)	12/50 (24%)	Repeat valve ablation (n = 12), bladder neck incision (n = 3)
Androulakakis 2005 [17]	18	2.4 (0.5–4)	112 (72–204)	N/A	4/18 (22%)	Repeat valve ablation
Emir 2002 [18]	26	54 (2–156)	66 (27–156)	N/A	5/26 (19%)	Bladder augmentation
Imaji 2002^c^ [25]	55	(0–180)	3	2/55 (3.6%)	18/55 (33%)	Repeat valve ablation (n = 18), dilatation (n = 2)
Podesta 2002 [10]	8	11 (1–35)	139 (72–216)	N/A	1/12 (12.5%)	Ureterreimplantation
Nonomura 1999 [13]	74	60 (3–192)	N/A	N/A	13/74 (18%)	Repeat valve ablation
Lal 1998 [24]	82	(12–180)	(12–252)	3/82 (3.6%)	N/A	N/A
Kurth 1981 [39]	125	(0–216)	N/A	2/125 (1.6%)	N/A	Urethrotomy

FU = follow up, N/A = not available.

a Standard voiding cystourethrography (VCUG) was done 1–3 months after endoscopic valve resection.

b Median.

c Standard repeat cystoscopy was done 3 months after endoscopic valve resection.

Additional surgery is described in seven studies. In one study repeat cystoscopy was standard procedure 3 months after first EVR, independent of the clinical course [25]. They defined patients with minor, moderate and severe membranous lesions in the posterior urethra, and repeated cystourethroscopy 3 months following fulguration. Over 47% (18 out of 38) of the patients who had a severe obstructing membrane needed further fulguration. In another study the decision to repeat valve ablation was based on results of VCUG performed in all patients 1 to 3 months post- treatment [26]. Five studies found that repeat valve ablation was necessary in 15 to 33% of the treated boys.

One study found that 19% of the treated boys needed bladder augmentation during follow-up. In another study ureteral reimplantation was performed in 1 out of 8 (12.5%) treated boys after EVR.

## Discussion

To our knowledge this is the first systematic review on the outcomes of endoscopic treatment of PUV. We found that the reported incidence of renal and bladder dysfunction in patients with PUV after endoscopic PUV treatment varies widely. This may reflect a broad clinical spectrum, which relates to the lack of a standardised quantification of obstruction and its severity. Moreover, methods and reporting of studies was not standardized. The risk of bias is rather high, and therefore we put little confidence in the reported estimates of effect. Apart from elevated nadir serum creatinine as the only predictor for long-term CKD or ESRD, we found no other specific characteristics which enables identification of subgroups. The reported data indicate that patients with PUV clearly are at risk for increased and chronic urodynamic abnormalities, i.e. PVR, and over time bladder hypocontractility and reduction in compliance, possibly resulting in more severe impairment of upper tracts.

However, drawing conclusions based on the data reported in this systematic review should be done with caution for the following reasons.

First of all, the available studies are large post surgery follow-up series at best, characterized by heterogeneous patients and data, and lacking standardized reporting, follow-up time and control groups.

Secondly, the broad clinical spectrum of reported outcomes complicated the description and comparison of studies. Given the high number of patients with severely compromised upper tracts, selective reporting of patients with more serious sequelae is rather likely. In our clinic patients tend to present at later age with incontinence and bladder dysfunction rather than upper tract deterioration, reflecting less severe infravesical obstruction. A cohort of late-diagnosed patients with PUV was described by Schober et al., they found that in most patients with normal imaging of the urinary tract presented with night time incontinence and frequency [12].

Thirdly, we used separate outcomes for upper and lower urinary tract function, although these outcomes may be interrelated in most of the cases. Boys with severe bladder dysfunction supposedly have the greatest risk for upper tract damage, although this could not be confirmed with our data. Moreover, there is evidence that bladder function changes over time, especially during puberty [20], [21], [23].

Fourth, many studies reported resolution of VUR as EVR outcome and the cumulative incidence of VUR varied widely across studies. However, VUR as an therapeutic outcome measure after EVR provides debatable evidence since in young children VUR may resolve spontaneously or with conservative measures and studies comparing surgery to conservative measures are lacking [27]. Fifth, the reported outcomes depend on multiple factors including age at presentation and the duration of follow-up. Both varied widely across studies and complicated the comparison of outcomes. Boys with severe sequelae of PUV tend to present early in childhood and may represent the more severe side of the PUV spectrum with higher risks of CKD and ESRD. Intense follow-up as standard care independent of the clinical course is important in this group. Boys presenting late in childhood with a urinary tract infection, incontinence or LUTS are likely to represent the mild end of the PUV spectrum, and less intensive follow-up is needed.

The degree of neonatal obstruction is not a predictive value on the final bladder function. A significant proportion of the children develop severe bladder dysfunction, often only years later. For these children, bladder dysfunction after PUV is a life-long illness, especially associated with CKD.

Finally, some studies report few patients with reoperation while others report all scheduled standard re-valve resections. A repeat valve ablation may mean that the obstruction was too severe to be solved in one procedure, or may be due adhesion of the wound bed because the urethral sphincter is closed more than 95% of the time. Considering other types of additional surgery; to date evidence is lacking from randomized studies showing that EVR or re-EVR may prevent the need for ureteral re-implantation or bladder augmentation. Few complications have been reported in the studies included; however it should be noted that follow-up in most studies is relatively short.

PUV are recognized since 1919 [28] and different classifications based on valve type and degree of obstruction have been described in literature [29]. Nowadays there is still no classification to distinguish between mild and severe disease. The available follow-up data clearly do not provide direct evidence of effectiveness of EVR, nor support of a tool to predict final outcome. These data may reflect regression to the mean, random error or the pathophysiology of infravesical obstruction in boys.

## Conclusion

The reported cumulative incidence renal and bladder dysfunction in patients with PUV after EVR treatment varies widely. We only identified nadir serum creatinine to be associated with renal dysfunction. Absence of evidence for other predictors may reflect the broad clinical spectrum, but at the same time the rather high risk of bias and the poor reporting of studies should be taken into account. Future studies into longer term follow-up of patients with PUV after EVR treatment should employ standardization of measurements and report on risk of bias. Moreover, the case mix and spectrum of posterior valve disease should be taken into account.

## Supporting Information

Table S1 **PRISMA 2009 checklist.**
(DOC)

Table S2 **Search strategy.**
(DOC)
