# Supplementary material for: A Systematic Review on Renal and Bladder Dysfunction after Endoscopic Treatment of Infravesical Obstruction in Boys
Source: PLoS One. 2012 Sep 13;7(9):e44663. doi: 10.1371/journal.pone.0044663 (PMC3441498; doi:10.1371/journal.pone.0044663)
Supplement: Table S2 — Search strategy. (DOC) [file pone.0044663.s002.doc]

**Table S2. Search Strategy**

*Objective:*

*((*obstruction[Title/Abstract] OR obstructions[Title/Abstract] OR obstruent[Title/Abstract] OR obstructive[Title/Abstract] OR obstructed[Title/Abstract] OR stricture[Title/Abstract] OR stenosis[Title/Abstract]) *AND* (infravesical[Title/Abstract] OR urethra[Title/Abstract] OR Urethral[Title/Abstract] OR Intraurethral[Title/Abstract] OR urinary tract[Title/Abstract] OR urinary tracts[Title/Abstract])) ***OR*** ((valve[Title/Abstract] OR valves[Title/Abstract]) *AND* (urethra[Title/Abstract] OR urethral[Title/Abstract] OR flap[Title/Abstract] OR infravesical[Title/Abstract])) **OR**(puv[Title/Abstract] OR mohrmann[Title/Abstract] OR diverticle[Title/Abstract] OR bladder neck[Title/Abstract])

*Treatment:*

(ablation[Title/Abstract] OR resection[Title/Abstract] OR surgery[Title/Abstract] OR surgical[Title/Abstract] OR surgically[Title/Abstract] OR ablative[Title/Abstract] OR dissection[Title/Abstract] OR fulguration[Title/Abstract] OR removal[Title/Abstract] OR incision[Title/Abstract] OR endoscopic[Title/Abstract])

*Children:*

(child[Title/Abstract] OR children[Title/Abstract] OR boy[Title/Abstract] OR boys[Title/Abstract] OR pediatric[Title/Abstract] OR pediatrics[Title/Abstract] OR paediatric[Title/Abstract] OR paediatrics[Title/Abstract] OR juvenile[Title/Abstract]) OR infant[Title/Abstract] OR neonate[Title/Abstract] OR neonatal[Title/Abstract])

**SEARCH: Objective AND Treatment AND Children**.
